# New Torsional Surface Elastic Waves in Cylindrical Metamaterial Waveguides for Sensing Applications

**DOI:** 10.3390/s25010143

**Published:** 2024-12-29

**Authors:** Piotr Kiełczyński, Krzysztof Wieja, Andrzej Balcerzak

**Affiliations:** Institute of Fundamental Technological Research, Polish Academy of Sciences, ul. Pawińskiego 5B, 02-106 Warsaw, Poland; kwieja@ippt.pan.pl (K.W.); abalcerz@ippt.pan.pl (A.B.)

**Keywords:** torsional elastic waves, elastic metamaterials, negative elastic compliance, dispersion curves, phase velocity, group velocity, mass sensitivity, viscosity sensors

## Abstract

In this paper, we demonstrate that torsional surface elastic waves can propagate along the curved surface of a metamaterial elastic rod (cylinder) embedded in a conventional elastic medium. The crucial parameter of the metamaterial rod is its elastic compliance $s_{44}^{\left(1\right)}\left(\omega \right),$ which varies as a function of frequency ω analogously to the dielectric function $\varepsilon \left(\omega \right)$ in Drude’s model of metals. As a consequence, the elastic compliance $s_{44}^{\left(1\right)}\left(\omega \right)$ can take negative values $s_{44}^{\left(1\right)}\left(\omega \right)<0$ as a function of frequency ω. Negative elastic compliance ($s_{44}^{\left(1\right)}\left(\omega \right)<0$) enables the emergence of new surface states, i.e., new types of surface elastic waves. In fact, the proposed torsional elastic surface waves can be considered as an elastic analog of Surface Plasmon Polariton (SPP) electromagnetic (optical) waves propagating along a metallic rod (cylinder) embedded in a dielectric medium. Consequently, we developed the corresponding analytical equations, for the dispersion relation and group velocity of the new torsional elastic surface wave. The newly discovered torsional elastic surface waves exhibit virtually all extraordinary properties of their electromagnetic SPP counterparts, such as strong subwavelength concentration of the wave energy in the vicinity of the cylindrical surface (r=a) of the guiding rod, very low phase and group velocities, etc. Therefore, the new torsional elastic surface waves can be used in: (a) near-field subwavelength acoustic imaging (super-resolution), (b) acoustic wave trapping (zero group and phase velocity), etc. Importantly, the newly discovered torsional elastic surface waves can form a basis for the development of a new generation of ultrasonic sensors (e.g., viscosity sensors), biosensors, and chemosensors with a very high mass sensitivity.

## 1. Introduction

We are currently witnessing a fascinating development of the theory of surface and bulk acoustic waves. New extraordinary properties in the domain of acoustic waves appeared with the invention of a new class of materials, i.e., metamaterials. The use of elastic metamaterials for the construction of ultrasonic waveguides has created a fertile ground for the discovery of a series of new ultrasonic waves.

As an example of newly discovered elastic surface waves propagating on a flat interface between two elastic half-spaces, one of which is an elastic metamaterial with a negative elastic compliance, can serve the SH surface acoustic waves discovered by Kiełczyński and presented in [1,2]. 

The search for new types of ultrasonic sensors led the authors of this paper to the discovery of new torsional elastic waves propagating on the curved surface of metamaterial elastic cylinders embedded in a conventional elastic medium. These newly discovered elastic torsional waves can be applied to develop a new generation of acoustic cylindrical sensors with very high mass sensitivity. Needless to say, such a property of the sensor is of crucial importance in measurements in many applications in domains, such as medicine, biology, toxicology, or environmental studies.

In this paper, the authors proved the existence of a new class of elastic torsional surface waves propagating in cylindrical waveguides with a metamaterial cylindrical rod, with a negative elastic compliance $s_{44}<0$, embedded in a conventional elastic medium with $s_{44}>0$. The newly discovered torsional elastic surface waves have only one angular (shear) mechanical displacement component $u_{\theta }$ that is tangential to the cylinder circumference and depends only on the radial coordinate r.

The curved cylindrical structure of the waveguide offers many advantages in practical field measurements, e.g., the cylindrical shape of the waveguide supporting the new torsional elastic surface waves can be beneficial in operation in a liquid environment. Simultaneously, we expect that the newly discovered torsional elastic surface waves, propagating along cylindrical rods, will exhibit very high mass sensitivity.

The main feature of the newly discovered torsional elastic surface waves is their close affinity with the Surface Plasmon Polariton (SPP) electromagnetic (optical) waves propagating along the interface between the metallic core cylinder and the dielectric outer medium [3,4]. In fact, the newly discovered torsional elastic surface waves can be considered as an elastic counterpart of the electromagnetic SPP waves described in [3,4].

Ultrasonic elastic waves propagating in pure elastic rectangular (flat) and circular waveguide structures have found applications in sensors of physical quantities, such as viscosity sensors, to investigate the elastic parameters of surface layers, to investigate the physicochemical parameters of liquids, etc. [5,6,7,8,9,10,11,12,13,14].

Torsional waves propagating in pure elastic cylindrical rods have been mainly used in viscosity sensors since the 1950s [15]. Classical ultrasonic cylindrical liquid viscosity sensors are made of conventional elastic materials. These sensors are usually used to determine the viscosity of liquids in biosensors and chemosensors [16,17,18,19,20,21].

The mechanical displacement of a torsional wave is tangential to the surface of the elastic cylinder in which the wave propagates. The cylindrical waveguide is immersed in a liquid whose material parameters (e.g., density, viscosity) are to be determined. Thus, the torsional wave generates a shear stress on the cylinder surface and in the surrounding investigated liquid. The amplitude and speed of propagation of torsional waves change as a result of the loading of the cylindrical surface by the adjacent liquid. This phenomenon can constitute the basis for the operation of viscosity sensors using elastic torsional waves.

However, sensors of this type are not free from disadvantages. The main inherent deficiency of torsional waveguides based on the use of conventional elastic materials is that the acoustic energy of the torsional wave is distributed over a large volume of the cylinder. For this reason, the energy density of the wave near the cylindrical surface is moderate. This results in the mass sensitivity of this torsional wave being moderate. In conventional torsional wave viscosity sensors, a larger concentration of the wave energy near the cylindrical surface is impossible due to the diffraction phenomenon. Therefore, the need to solve this problem arose.

The aim of the authors’ work was to overcome these drawbacks. To solve this problem, the authors used the extraordinary properties of elastic metamaterials with a negative elastic compliance $s_{44}<0$.

Employment of the elastic metamaterials was inspired by the fact that the use of mechanical and optical metamaterials for the construction of the sensors, chemosensors, and biosensors substantially improved their exploitation characteristics [22,23,24,25,26]. Namely, optical Surface Plasmon Polariton (SPP) waves propagating at the interface between a metallic half-space and a dielectric half-space are characterized by a large concentration of wave energy near the metal-dielectric interface [27]. A similar large concentration of wave energy occurs in the case of optical SPP waves propagating in cylindrical waveguides (a metallic cylinder embedded in a conventional dielectric material) [4].

Using analogies between SPP-type electromagnetic waves and Shear Horizontal (SH) elastic waves, a new SH-type elastic wave propagating at a flat interface between two elastic half-spaces, one of which is a metamaterial half-space ($s_{44}<0$), was discovered by Kiełczyński in [2]. This new elastic surface wave is also characterized by a huge concentration of wave energy near the interface.

These facts motivated the authors to search for new elastic torsional waves propagating on the surface of metamaterial cylindrical waveguides. In this paper, the authors describe a newly discovered torsional elastic wave propagating on the surface of a metamaterial cylinder ($s_{44}<0$) embedded in a conventional elastic material ($s_{44}>0$).

According to the analysis carried out by the authors in this paper, this newly discovered torsional wave is characterized by a very high concentration of elastic wave energy near the cylinder surface, which should also result in a very large increase in the mass sensitivity of the new torsional wave.

We can treat this newly discovered elastic torsional wave as an elastic analog of optical (electromagnetic) SPP waves propagating on the surface of a metallic cylinder embedded in a conventional dielectric material described in [4].

**Figure 1:**
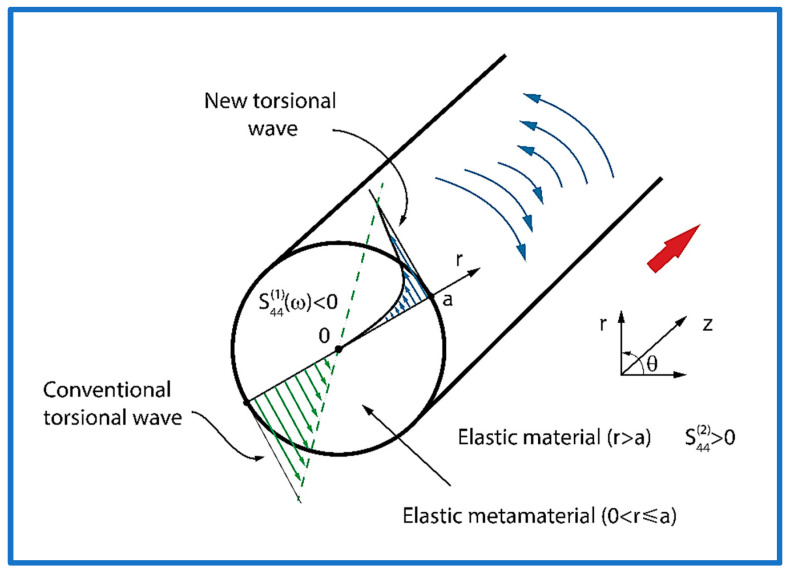
Cross-section of the cylindrical waveguide supporting the new torsional elastic surface waves, propagating along the cylindrical metamaterial rod (0<r≤a), embedded in a conventional elastic medium (r>a). Mechanical displacement $u_{\theta }$ of the new torsional elastic surface wave is polarized along the angular coordinate θ. The red arrow indicates the direction of propagation. The dashed (green) lines specify the mechanical displacement of the conventional torsional waves propagating in a purely elastic cylindrical waveguide. The blue arrows point out the mechanical displacement of the new surface elastic torsional wave propagating in the elastic metamaterial cylindrical waveguide.

The equations of motion written in a cylindrical coordinate system were formulated and solved. Analytical formulas of (1) the dispersion equation and (2) group velocity were developed for the elastic torsional wave propagating in layered cylindrical metamaterial structures in the *z*-axis direction (see Figure 1).

The key property of the newly discovered torsional ultrasonic waves is that their mechanical displacement is concentrated close to the curved surface (r=a) of the cylinder, which greatly increases the mass sensitivity of the sensors that use the newly discovered torsional waves.

It should be finally mentioned that the proposed newly discovered torsional elastic surface waves exhibit virtually all extraordinary properties of their electromagnetic SPP counterparts, such as (1) strong subwavelength concentration of energy in the vicinity of the curved cylindrical surface of the guiding rod, (2) very low phase and group velocities, (3) subwavelength penetration depth, (4) possibility to achieve a resolution below a wavelength (super resolution), etc.

The layout of this paper is as follows. After the introduction in Section 1, we present the geometrical structure and material parameters of the cylindrical metamaterial waveguide supporting the new torsional elastic surface waves in Section 2. In Section 3, we develop mathematical equations for the mechanical displacement, shear stresses, dispersion relation, and group velocity for the new torsional elastic surface waves. Section 4 contains numerical results and figures resulting from the analysis performed in Section 3. Section 5 and 6 contain the discussion and conclusions, respectively.

## 2. Physical Model

### 2.1. Geometry and Material Parameters of the Waveguide

The geometry of the waveguide supporting the new torsional elastic surface waves is presented in Figure 1. The waveguide consists of a metamaterial elastic cylindrical rod (0<r≤a) embedded in a conventional elastic medium (r>a). As will be shown explicitly in Section 2.2, the elastic compliance of the metamaterial cylindrical rod (0<r≤a) can exhibit negative values $s_{44}^{\left(1\right)}\left(\omega \right)<0$ in the frequency range adjacent to zero frequency ω=0. The remaining material parameters of the waveguide, i.e., the density in the metamaterial rod $\rho_{1}$ and in a conventional elastic surrounding medium $\rho_{2}$, as well as its elastic compliance $s_{44}^{\left(2\right)}>0$, are all positive.

### 2.2. Elastic Compliance $s_{44}^{\left(1\right)}\left(\omega \right)$ of the Metamaterial Elastic Cylinder 0<r≤a

It is assumed throughout this paper that the elastic compliance $s_{44}^{\left(1\right)}\left(\omega \right)$ of the metamaterial cylinder (rod), as a function of angular frequency ω, changes analogously to the dielectric function $\varepsilon \left(\omega \right)$ in Drude’s model of metals [28], namely

(1) $$s_{44}^{\left(1\right)}(\omega )=s_{0}\left(1-\frac{\omega_{p}^{2}}{\omega^{2}}\right)$$

where $\omega_{p}$ is the angular frequency of the local mechanical resonators in the metamaterial and $s_{0}$ is its reference elastic compliance for ω→∞.

In Drude’s model of metals the angular frequency, $\omega_{p}=2\pi f_{p}$ is called the angular frequency of bulk plasmon resonance [27]. The adjacent medium (r>a) is a conventional elastic material with a positive compliance $s_{44}^{\left(2\right)}>0$ and density $\rho_{2}>0$ that are both frequency independent.

The physical mechanism that explains the fact that the dielectric constant ε in the Drude model takes negative values is that the oscillations of free electrons in the metal are delayed in phase by 180° with respect to the phase of the driving electric field. Similarly, in the case of elastic metamaterials, the vibrations of the local mechanical oscillators are also delayed in phase by 180° relative to the phase of the mechanical displacement of the driving transverse ultrasonic wave. In this way, the elastic mechanical compliance $s_{44}$ can take negative values.

The vibrations of local electrical resonators and local mechanical resonators are described by the same mathematical model. Therefore, the Drude formula, which describes the vibrational properties of electrical resonators, can also be used to characterize the vibrational parameters of mechanical resonators (e.g., mechanical admittance and impedance).

It should be stressed that according to Equation (1) the elastic compliance $s_{44}^{\left(1\right)}\left(\omega \right)$ of the metamaterial rod is negative in the frequency range $0<\omega <\omega_{p}$. How to realize the elastic metamaterial with a negative elastic compliance $s_{44}^{\left(1\right)}\left(\omega \right)<0$ was shown in the recent paper of Kiełczyński [2] in Sections 2.3 and 2.4.

## 3. Mathematical Model

A unique feature of the new torsional elastic surface waves is the fact that they possess only one component of the mechanical displacement $u_{\theta }$ that is polarized along the angular coordinate θ, which is tangential to the circumference of the cylinder (see Figure 1).

The mechanical displacement of the new torsional elastic surface wave decays rapidly with the distance from the surface of the cylinder (r=a) in both directions, i.e., into the metamaterial elastic cylinder (0<r≤a) and into the adjacent elastic medium (r>a).

### 3.1. Mechanical Displacement and Shear Stress

The new torsional elastic surface wave propagates along the axis of the cylinder z. The mechanical displacement $u_{\theta }^{\left(1\right)}(r,z,t)$ of the new torsional elastic surface wave in the metamaterial elastic cylinder 0<r≤a can be expressed in the following generic form:

(2) $$u_{\theta }^{\left(1\right)}(r,z,t)=A\cdot f(r)\cdot exp\left[j\left(kz-\omega t\right)\right]$$

where the function f(r) depends only on the radial distance r, $j=\sqrt{-1}$ stands for the imaginary unit, k is the wavenumber of the new torsional elastic surface wave, ω is its angular frequency, A is an arbitrary real constant, and t stands for time.

By definition, the associated shear stress $\sigma_{r\theta }^{\left(1\right)}(r,z,t)$ of the new torsional elastic surface wave in the metamaterial elastic cylinder 0<r≤a is given by

(3) $$\sigma_{r\theta }^{\left(1\right)}(r,z,t)=\frac{1}{s_{44}^{\left(1\right)}\left(\omega \right)}r\frac{\partial }{\partial r}\left(\frac{u_{\theta }^{\left(1\right)}(r,z,t)}{r}\right)$$

Analogous expressions can be written for the mechanical displacement $u_{\theta }^{\left(2\right)}(r,z,t)$ and the associated shear stress $\sigma_{r\theta }^{\left(2\right)}(r,z,t)$ in the adjacent conventional elastic medium (r>a), namely

(4) $$u_{\theta }^{\left(2\right)}(r,z,t)=B\cdot g(r)\cdot exp\left[j\left(kz-\omega t\right)\right]$$

and

(5) $$\sigma_{r\theta }^{\left(2\right)}(r,z,t)=\frac{1}{s_{44}^{\left(2\right)}}r\frac{\partial }{\partial r}\left(\frac{u_{\theta }^{\left(2\right)}(r,z,t)}{r}\right)$$

where the function g(r) depends only on the radial distance r, and B is an arbitrary real constant.

The functions f(r) and g(r) with a radial argument r describe the change in the amplitude of the new torsional elastic surface wave inside the cylindrical metamaterial rod and in the surrounding medium, respectively.

The functions f(r) and g(r) will be given in a closed analytical form by Equations (10) and (11) and the wavenumber k will be determined from the dispersion relation Equation (14) in Section 3.5 of this paper.

### 3.2. Equations of Motion

The mechanical displacements of the new torsional elastic surface wave: in the metamaterial elastic rod $u_{\theta }^{\left(1\right)}$ and in the adjacent conventional elastic medium $u_{\theta }^{\left(2\right)}$ satisfy the following equations of motion, written in the cylindrical system of coordinates:

(6) $$\rho_{1}s_{44}^{\left(1\right)}\frac{\partial^{2}u_{\theta }^{\left(1\right)}}{\partial t^{2}}=\frac{1}{r^{2}}\cdot \frac{\partial }{\partial r}\left(r^{3}\frac{1}{s_{44}^{\left(1\right)}}\frac{\partial }{\partial r}\left(\frac{u_{\theta }^{\left(1\right)}}{r}\right)\right)+\frac{\partial }{\partial z}\left(\frac{1}{s_{44}^{\left(1\right)}}\cdot \frac{\partial u_{\theta }^{\left(1\right)}}{\partial z}\right)\;0<r\leq a$$

and

(7) $$\rho_{2}s_{44}^{\left(2\right)}\frac{\partial^{2}u_{\theta }^{\left(2\right)}}{\partial t^{2}}=\frac{1}{r^{2}}\cdot \frac{\partial }{\partial r}\left(r^{3}\frac{1}{s_{44}^{\left(2\right)}}\frac{\partial }{\partial r}\left(\frac{u_{\theta }^{\left(2\right)}}{r}\right)\right)+\frac{\partial }{\partial z}\left(\frac{1}{s_{44}^{\left(2\right)}}\cdot \frac{\partial u_{\theta }^{\left(2\right)}}{\partial z}\right)\;\;\;\;r>a$$

where, for the sake of clarity, the arguments in the mechanical displacements $u_{\theta }^{\left(1\right)}\;and\;u_{\theta }^{\left(2\right)}$ were omitted.

### 3.3. Explicit Analytical Formulas for the Mechanical Displacements $u_{\theta }^{\left(1\right)}$ and $u_{\theta }^{\left(2\right)}$

Substituting Equation (2) for the mechanical displacement $u_{\theta }^{\left(1\right)}$ in the metamaterial rod into the equation of motion Equation (6), we obtain an ordinary differential equation for the unknown radial function f(r). It can be shown that the solution for this differential equation takes the following form:

(8) $$f\left(r\right)=I_{1}\left(\gamma_{1}r\right)$$

where $\gamma_{1}$ is the radial wavenumber $\gamma_{1}={\left[k^{2}-\omega^{2}\rho_{1}s_{44}^{\left(1\right)}(\omega )\right]}^{1/2}$ and $I_{1}$ stands for the modified Bessel function of the first kind of order 1.

Similarly, substituting Equation (4) for the mechanical displacement $u_{\theta }^{\left(2\right)}$ in the adjacent medium into the equation of motion Equation (7), we obtain an ordinary differential equation for the unknown radial function g(r), whose solution reads

(9) $$g\left(r\right)=K_{1}\left(\gamma_{2}r\right)$$

where $\gamma_{2}$ is the radial wavenumber $\gamma_{2}={\left(k^{2}-\omega^{2}\rho_{2}s_{44}^{\left(2\right)}\right)}^{1/2}$ and $K_{1}$ stands for the modified Bessel function of the second kind of order 1.

Finally, substituting Equation (8) into Equations (2) and (9) into Equation (4), one obtains

(10) $$u_{\theta }^{\left(1\right)}\left(r,z,t\right)=A\cdot I_{1}\left(\gamma_{1}r\right)\cdot exp\left[j\left(kz-\omega t\right)\right]\;\;\;\;0<r\leq a$$

(11) $$u_{\theta }^{\left(2\right)}\left(r,z,t\right)=B\cdot K_{1}\left(\gamma_{2}r\right)\cdot exp\left[j\left(kz-\omega t\right)\right]\;\;\;\;\;r>a$$

### 3.4. Boundary Conditions

The mechanical displacement $u_{\theta }$ and shear stress $\sigma_{r\theta }$ of the new torsional elastic surface wave must be continuous across the surface of the cylindrical surface r=a of the metamaterial rod, i.e.,

(12) $${\left.u_{\theta }^{\left(1\right)}\right|}_{r=a}={\left.u_{\theta }^{\left(2\right)}\right|}_{r=a}$$

(13) $${\left.\sigma_{r\theta }^{\left(1\right)}\right|}_{r=a}={\left.\sigma_{r\theta }^{\left(2\right)}\right|}_{r=a}$$

### 3.5. Dispersion Equation

In the first step in determination of the dispersion equation for the new torsional elastic surface waves, we will substitute Equations (2)–(5) into the boundary conditions Equations (12) and (13). As a result, we will obtain a system of two linear homogeneous algebraic equations for the unknown constants A and B. Equating the determinant of this system of equations to zero, we obtain the following dispersion equation of the new torsional elastic surface waves:

(14) $$\frac{K_{2}\left(\gamma_{2}a\right)}{K_{1}\left(\gamma_{2}a\right)}+\frac{s_{44}^{\left(2\right)}}{s_{44}^{\left(1\right)}\left(\omega \right)}\frac{\gamma_{1}}{\gamma_{2}}\frac{I_{2}\left(\gamma_{1}a\right)}{I_{1}\left(\gamma_{1}a\right)}=F\left(\omega ,\;k\right)=0$$

where $I_{1}$ and $I_{2}$ are the modified Bessel functions of the first kind of order 1 and 2, and similarly, $K_{1}$ and $K_{2}$ are the modified Bessel functions of the second kind of order 1 and 2.

The dispersion relation Equation (14) is a transcendental nonlinear algebraic equation for the wavenumber k at a fixed angular frequency ω, which can be solved numerically using appropriate numerical procedures, such as, e.g., the iterative Newton–Raphson method.

### 3.6. Group Velocity

Group velocity $v_{gr}\left(\omega \right)$ of the new torsional elastic surface waves was evaluated analytically using the following formula: $v_{gr}\left(\omega \right)=-\frac{\partial F\left(\omega ,\;k\right)/\partial k}{\partial F\left(\omega ,\;k\right)/\partial \omega }$, where the function $F\left(\omega ,\;k\right)$ represents the dispersion equation (see Equation (14)), which is obviously an implicit function of the angular frequency ω and wavenumber k. Thus, after lengthy but quite elementary algebra, we obtain

(15) $$v_{gr}=-\frac{\begin{array}{c} \left[s_{44}^{\left(1\right)}\left(\omega \right)\frac{\partial \gamma_{2}}{\partial k}+s_{44}^{\left(1\right)}\left(\omega \right)\left(\frac{\partial \gamma_{1}}{\partial k}\frac{\gamma_{2}}{\gamma_{1}}-2\frac{\partial \gamma_{2}}{\partial k}\right)\right]\frac{K_{2}\left(\gamma_{2}a\right)}{K_{1}\left(\gamma_{2}a\right)}+\left[s_{44}^{\left(2\right)}\frac{\partial \gamma_{1}}{\partial k}+s_{44}^{\left(2\right)}\left(\frac{\partial \gamma_{2}}{\partial k}\frac{\gamma_{1}}{\gamma_{2}}-2\frac{\partial \gamma_{1}}{\partial k}\right)\right]\frac{I_{2}\left(\gamma_{1}a\right)}{I_{1}\left(\gamma_{1}a\right)}+\left[s_{44}^{\left(1\right)}\left(\omega \right)\gamma_{2}a\frac{\partial \gamma_{1}}{\partial k}-s_{44}^{\left(2\right)}\gamma_{1}a\frac{\partial \gamma_{2}}{\partial k}\right]\frac{I_{2}\left(\gamma_{1}a\right)}{I_{1}\left(\gamma_{1}a\right)}\frac{K_{2}\left(\gamma_{2}a\right)}{K_{1}\left(\gamma_{2}a\right)}-\left[s_{44}^{\left(1\right)}\left(\omega \right)\gamma_{2}a\frac{\partial \gamma_{2}}{\partial k}-s_{44}^{\left(2\right)}\gamma_{1}a\frac{\partial \gamma_{1}}{\partial k}\right] \end{array}}{\left[{\frac{\partial s_{44}^{\left(1\right)}\left(\omega \right)}{\partial \omega }\gamma_{2}+s}_{44}^{\left(1\right)}\left(\omega \right)\frac{\partial \gamma_{2}}{\partial \omega }+s_{44}^{\left(1\right)}\left(\omega \right)\left(\frac{\partial \gamma_{1}}{\partial \omega }\frac{\gamma_{2}}{\gamma_{1}}-2\frac{\partial \gamma_{2}}{\partial \omega }\right)\right]\frac{K_{2}\left(\gamma_{2}a\right)}{K_{1}\left(\gamma_{2}a\right)}+\left[s_{44}^{\left(2\right)}\frac{\partial \gamma_{1}}{\partial \omega }+s_{44}^{\left(2\right)}\left(\frac{\partial \gamma_{2}}{\partial \omega }\frac{\gamma_{1}}{\gamma_{2}}-2\frac{\partial \gamma_{1}}{\partial \omega }\right)\right]\frac{I_{2}\left(\gamma_{1}a\right)}{I_{1}\left(\gamma_{1}a\right)}+\left[s_{44}^{\left(1\right)}\left(\omega \right)\gamma_{2}a\frac{\partial \gamma_{1}}{\partial \omega }-s_{44}^{\left(2\right)}\gamma_{1}a\frac{\partial \gamma_{2}}{\partial \omega }\right]\frac{I_{2}\left(\gamma_{1}a\right)}{I_{1}\left(\gamma_{1}a\right)}\frac{K_{2}\left(\gamma_{2}a\right)}{K_{1}\left(\gamma_{2}a\right)}-\left[s_{44}^{\left(1\right)}\left(\omega \right)\gamma_{2}a\frac{\partial \gamma_{2}}{\partial \omega }-s_{44}^{\left(2\right)}\gamma_{1}a\frac{\partial \gamma_{1}}{\partial \omega }\right]}$$

where $\frac{\partial \gamma_{1}}{\partial k}=\frac{k}{\gamma_{1}}$; $\frac{\partial \gamma_{2}}{\partial k}=\frac{k}{\gamma_{2}}$; $\frac{\partial \gamma_{1}}{\partial \omega }=\frac{-\rho_{1}\left(\omega s_{44}^{\left(1\right)}\left(\omega \right)+s_{0}\omega_{p}^{2}/\omega \right)}{\gamma_{1}}$; $\frac{\partial \gamma_{2}}{\partial \omega }=\frac{-\omega \rho_{2}s_{44}^{\left(2\right)}}{\gamma_{2}}$ and $\frac{\partial s_{44}^{\left(1\right)}\left(\omega \right)}{\partial \omega }=2s_{0}\omega_{p}^{2}/\omega^{3}$.

At first glance, Equation (15) looks lengthy and intimidating, with doubtful operational significance; however, it can be easily implemented in numerical calculations using standard procedures from software packages, such as Scilab or Matlab.

The computer program written in Scilab programming language to solve the dispersion equation (Equation (14)) to evaluate the dispersion curve and calculate the group velocity (Equation (15)) is given in Appendix A.

## 4. Results of Numerical Calculations

### 4.1. Material Parameters of the Waveguide

Numerical calculations were performed employing an exemplary waveguide structure consisting of a metamaterial cylindrical rod (0<r≤a) made of ST-Quartz with embedded local oscillators and PMMA surrounding medium (r>a). We assume that the frequency of the local elementary oscillators equals $f_{p}=1\;MHz$. The radius of the metamaterial cylindrical rod is a=1 cm. Losses in the cylindrical waveguide structure are neglected. The actual values of the material parameters used in the numerical calculations are given in Table 1.

Numerical calculations were performed with the help of the Scilab software package.

### 4.2. Dispersion Curve

**Figure 2:**
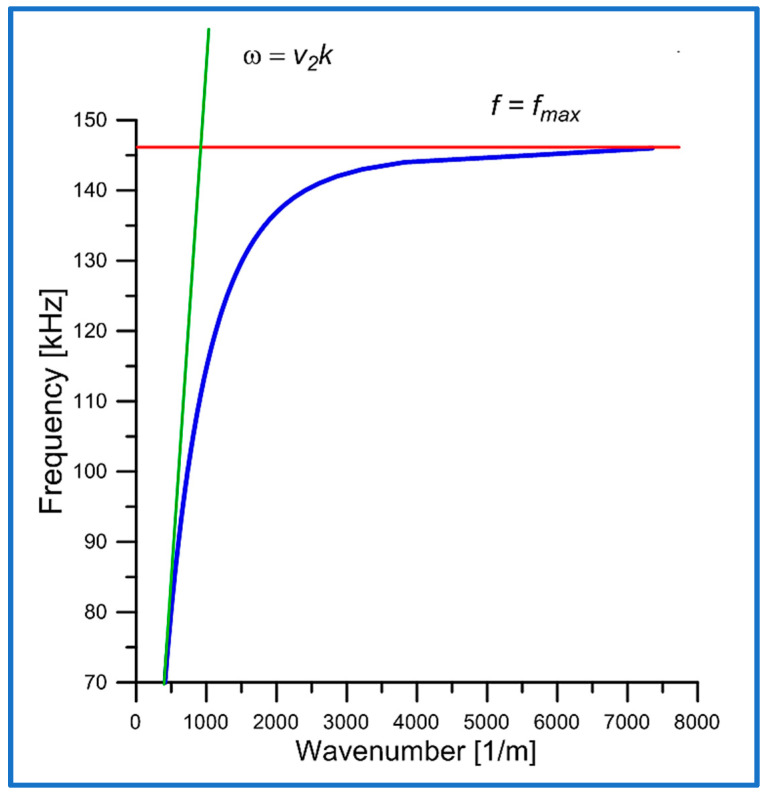
Dispersion curve (blue color) of the new torsional elastic surface wave, as the wave frequency f versus the wavenumber k. The green line shows the dispersion curve of bulk shear waves in a surrounding conventional elastic material.

The dispersion curve of the new torsional elastic surface wave was calculated from the solution of the dispersion relation Equation (14) and plotted in Figure 2 as the wave frequency f versus the wave number k. The phase velocity of the bulk shear elastic waves in the surrounding conventional elastic medium is denoted in Figure 2 as $v_{2}={\left(\rho_{2}s_{44}^{\left(2\right)}\right)}^{-1/2}$. The surface resonant frequency is an upper cut-off frequency since above this frequency the new torsional elastic surface wave cannot propagate.

The new torsional wave is a surface wave. From the analysis of the dispersion Equation (14), it results that a new torsional wave can only exist in the range $\left(\omega_{min},\omega_{max}\right)$. The lower limit $\omega_{min}$ is defined by the cylinder curvature. Above the upper $\omega_{max},$ the torsional wave cannot be a surface wave. In this frequency range $\left(\omega_{min},\omega_{max}\right),$ the phase velocity $v_{p}$ and the group velocity $v_{gr}$ tend to zero as $\omega \to \omega_{max}$.

### 4.3. Phase Velocity

**Figure 3:**
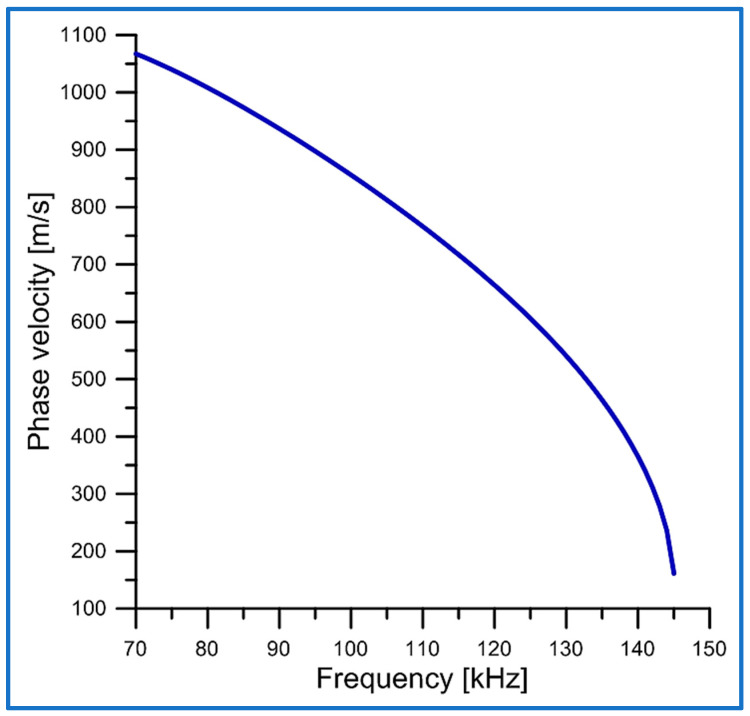
Phase velocity $v_{p}$ of the new torsional elastic surface wave versus frequency f. The lower cut-off frequency equals approximately 70 KHz.

Using the solution of the dispersion Equation (14), the plot of the phase velocity *v*_*p*_(*ω*) = *ω*/*k* as a function of the wave frequency f was evaluated and is presented in Figure 3. In our calculations, the maximum frequency $f_{max}$ is equal to approximately 146 kHz.

### 4.4. Group Velocity

**Figure 4:**
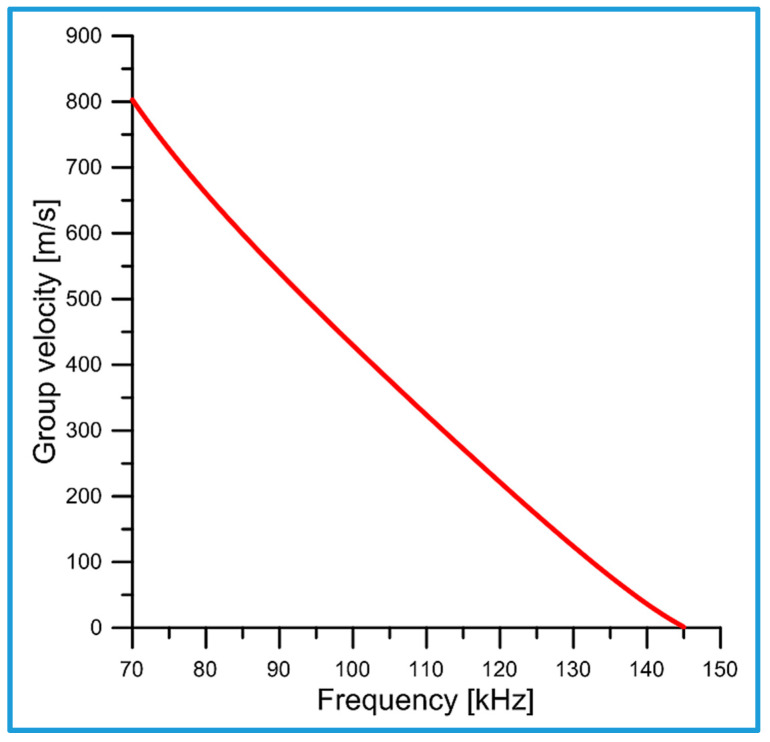
Group velocity $v_{gr}$ of the new torsional elastic surface wave versus frequency f.

Figure 4 shows the plot of the group velocity $v_{gr}\left(\omega \right)$ of the newly discovered torsional elastic surface wave as a function of the wave frequency f. The numerical calculations were performed using an analytical formula, Equation (15).

Note that group velocity $v_{gr}$ of the new torsional elastic surface wave is always lower than its phase velocity $v_{p},$ as shown in Figure 3, but has the same cut-off frequencies.

## 5. Discussion

### 5.1. Group Velocity

As the wave frequency grows, an increasing fraction of the elastic torsional wave power flows in the metamaterial elastic core. The torsional wave power in a conventional elastic medium ($s_{44}>0\;\mathrm{and}\;r>a$) flows in the opposite direction to the wave power flowing in the metamaterial elastic medium ($s_{44}<0\;\mathrm{and}\;r\leq a$). This reduces the overall power flow of the wave $P_{1}$ in the direction of propagation. By definition, energy velocity $v_{e}=P_{1}/u$, where: $P_{1}$ is the time-averaged total power flow in the propagation direction, and u is the time-averaged energy stored in the waveguide per unit length. Consequently, the energy velocity $v_{e}$ of the torsional wave decreases with increasing frequency.

Since the group velocity $v_{gr}$ can be identified with the energy flow velocity $v_{e}$, a decrease in the energy flow velocity $v_{e}$ entails a decrease in the group velocity of the wave $v_{gr}\to 0$ for $\omega \to \omega_{max}$. Moreover, it can be proven that the group velocity $v_{gr}$ is always smaller than the phase velocity ($v_{gr}<v_{p}$). Namely,

(16) $$v_{gr}=\frac{d\omega }{dk}=v_{p}+k\cdot \frac{dv_{p}}{dk}$$

Since in general $\frac{dv_{p}}{dk}<0$ (normal dispersion); therefore, $v_{gr}<v_{p}$.

This can be seen in Figure 3 and Figure 4.

### 5.2. Phase Velocity

The phase velocity $v_{p}$ behaves similarly to the group velocity $v_{gr}$.

Namely, $v_{p}=\omega /k$.

(17) $$\frac{dv_{p}}{d\omega }=\frac{1}{k}\cdot \left(1-\frac{v_{p}}{v_{gr}}\right)$$

Since $v_{gr}<v_{p}$, then $\frac{dv_{p}}{d\omega }<0$, i.e., the phase velocity $v_{p}$ decreases with increasing angular frequency ω. 

### 5.3. Dispersion Curve

The characteristic feature of the dispersion curve (see Figure 2) of the newly discovered torsional wave is that as the wave frequency f grows and approaches the cut-off frequency $f_{max}$, the wavenumber k increases significantly. Consequently, the wavelength λ=2π/k of the wave decreases and can reach the subwavelength region. This feature is responsible for the increase in the concentration of wave energy near the surface of the cylinder r=a and, therefore, for a substantial increase in the mass sensitivity of the new torsional wave.

Since the group velocity is, by definition, the derivative of the angular frequency ω with respect to the wavenumber k, then the dispersion curve, i.e., the plot of ω versus k, must decrease its slope as the angular frequency ω increases (see Figure 2).

In the limiting case $\omega \to \omega_{max}$, the group velocity $v_{gr}\to 0$ and the wavenumber k becomes very large k→∞.

The quest for sensors with enhanced parameters, such as very high sensitivity or very low detection threshold, is driven by the requirements resulting from numerous applications in medicine, biology, environmental studies, toxicology, etc. In fact, early detection of harmful bacteria, viruses, or toxins requires the development of appropriate sensors with a very high sensitivity and very low threshold of detection.

These important goals can be achieved using in general two different ways: first, by improvement of the existing sensors and technologies and second, by employment of new concepts, new types of waves, or new materials. Evidently, the second approach offers a chance to develop new revolutionary solutions with sensors of extraordinary parameters. However, it requires the navigation on uncharted waters, which may be not only very difficult but very often disappointing.

In this paper, we adhere to the second approach. In fact, in order to develop sensors with a very high mass sensitivity we propose to use the new type of elastic torsional surface waves which were discovered recently by the authors. These newly discovered elastic torsional waves propagate in the vicinity of the curved surface of the metamaterial cylindrical rod in which the elastic compliance $s_{44}^{\left(1\right)}\left(\omega \right)$ is analogous to the dielectric function $\varepsilon \left(\omega \right)$ in Drude’s model of metals.

Our choice of the new type of torsional elastic surface waves, propagating in metamaterial waveguides, can be justified by their extraordinary properties, which cannot be found in the existing elastic surface waves propagating in conventional pure elastic waveguides. For example, the newly discovered torsional elastic surface waves propagating along metamaterial cylinders (rods) exhibit the following unique properties:

1. Very high concentration of the wave energy in the vicinity of the cylindrical guiding surface (r=a) of the waveguide;
2. Subwavelength penetration depth in both directions from the cylindrical guiding surface (r=a);
3. Very low phase and group velocities (see Figure 3 and Figure 4).

As a matter of fact, all the above characteristics of the new torsional elastic surface waves can be employed in the development of ultrasonic sensors with a very high mass sensitivity. In addition, the cylindrical shape of the waveguide supporting the new torsional elastic surface waves can be advantageous in operations in a liquid environment.

## 6. Conclusions

In this paper, we discovered and presented new torsional elastic surface waves that propagate along elastic metamaterial rods (cylinders) embedded in a conventional elastic medium. The new torsional elastic surface waves have the following unique properties:

1. They constitute an elastic analog of the Surface Plasmon Polariton (SPP) electromagnetic (optical) waves propagating in layered dielectric-metal cylindrical waveguides;
2. New torsional elastic waves can inherit fascinating properties of SPP optical waves, such as (a) superlensing, (b) superresolution, and (c) the ability to break the diffraction limit;
3. They have only one component of the mechanical displacement polarized along the angular coordinate;
4. The energy of the wave is strongly confined in the vicinity of the guiding cylindrical surface (*r* = *a*) of the metamaterial rod;
5. The penetration depth of the wave in both directions from the guiding cylindrical surface of the metamaterial rod can be a subwavelength;
6. Their phase and group velocities tend to zero as the wave frequency approaches the upper cut-off frequency.

Consequently, due to their unique properties, which are presented above, the newly discovered torsional elastic surface waves analyzed in this paper have significant potential for the development of a new generation of ultrasonic sensors, biosensors, and chemosensors with a very high mass sensitivity for applications in medicine, biology, chemistry, and environmental research.

This work has an interdisciplinary character and, therefore, can be of interest to a wide range of researchers and engineers working in different domains of science and technology, such as acoustics, ultrasonics, optics, physics, microwaves, elastic metamaterials, ultrasonic sensors, biosensors, and chemosensors.

## Figures and Tables

**Table 1:** Material parameters of the cylindrical rod (ST-Quartz) and the adjacent (PMMA) medium. $s_{0}=s_{44}^{\left(1\right)}\left(\omega \to \infty \right)$, see Equation (1), phase velocity $v_{0}=\sqrt{1/\left(s_{0}\rho_{1}\right)}$.

Material	Density [kg/m^3^]	Elastic Compliance [GPa]	Bulk Shear Wave Velocity [m/s]
ST-Quartz	*ρ*_1_ = 2650	*s*_0_ = 1.474	*v*_0_ = 5060
PMMA	*ρ*_2_ = 1180	$s_{44}^{\left(2\right)}$ = 70.03	*v*_2_ = 1100

## Data Availability

The data is within the article.

## Appendix A

A computer program written in Scilab programming language to solve the dispersion equation (Equation (14)), evaluate the dispersion curve, and calculate the group velocity (Equation (15)) is given below. *//THIS PROGRAM SOLVES THE DISPERSION EQUATION* *//AND EVALUATES THE PHASE AND GROUP VELOCITIES* *//OF THE NEW ELASTIC TORSIONAL WAVE* *//MATERIAL DATA* c442 = 1.43*10^9 /*/PMMA = CONVENTIONAL ELASTIC MEDIUM* s442 = 1/c442 ro2 = 1.18*10^3 v2 = sqrt(c442/ro2) a = 1*10^-2 /*/RADIUS OF THE METAMATERIAL CYLINDER IN [m]* c0 = 6.785*10^10 /*/QUARTZ=METAMATERIAL CYLINDER* s0 = 1/c0 ro1 = 2.65*10^3 v0 = sqrt(c0/ro1) fp = 1*10^6 /*/FREQUENCY OF LOCAL RESONATORS* omp = 2*%pi*fp /*/ANGULAR FREQUENCY OF LOCAL RESONATORS* *//THIS FUNCTION CALCULATES THE ELASTIC COMPLIANCE s441* *//OF THE METAMATERIAL CYLINDER FOR GIVEN ANGULAR FREQUENCY om, EQ.1* function **F** = s441(**x**) om = **x** X1 = s0*(1-(omp/om)^2) **F** = X1 endfunction *//THIS FUNCTION EVALUATES THE DISPERSION EQUATION FOR THE TORSIONAL WAVE, EQ.14* *//FOR KNOWN ANGULAR FREQUENCY om AND THE WAVENUMBER k* function **F** = T_Dysp_2(**z**) k = **z** gamma1 = sqrt(k*k-om*om*ro1*s441(om)) gamma2 = sqrt(k*k-om*om*ro2*s442) x = gamma1*a y = gamma2*a *// besseli(2,x) and besseli(1,x) ARE MODIFIED BESSEL FUNCTIONS* *// OF THE FIRST KIND OF ORDER 2 AND 1* *// besselk(2,y) and besselk(1,y) ARE MODIFIED BESSEL FUNCTIONS* *// OF THE SECOND KIND OF ORDER 2 AND 1* X1 = besseli(2,x)/besseli(1,x) X2 = besselk(2,y)/besselk(1,y) X3 = s442/s441(om)*gamma1/gamma2 **F** = X2+X3*X1 endfunction *//THIS FUNCTION EVALUATES THE GROUP VELOCITY FOR THE TORSIONAL WAVE, EQ.15* function **G** = Group_Vel_2(**x**,**y**) om = **x** k = **y** gamma1 = sqrt(k*k-om*om*ro1*s441(om)) gamma2 = sqrt(k*k-om*om*ro2*s442) X = gamma1*a Y = gamma2*a dg1dk = k/gamma1 dg2dk = k/gamma2 dg1dom = (-om*ro1*s441(om)-ro1*s0*omp*omp/om)/gamma1 dg2dom = (-om*ro2*s442)/gamma2 *// DERIVATIVE OF THE ELASTIC COMPLIANCE S441 ON THE ANGULAR FREQUENCY om* ds441dom = 2*s0*omp*omp/om^3 L1 = s441(om)*dg2dk+s441(om)*Y*(dg1dk/X-dg2dk*2/Y) L2 = s442*dg1dk-s442*X*(dg1dk*2/X-dg2dk/Y) L3 = -s441(om)*Y*dg2dk+s442*X*dg1dk L4 = s441(om)*Y*dg1dk-s442*X*dg2dk M1 = ds441dom*gamma2+s441(om)*dg2dom+s441(om)*Y*(dg1dom/X-dg2dom*2/Y) M2 = s442*dg1dom-s442*X*(dg1dom*2/X-dg2dom/Y) M3 = -s441(om)*Y*dg2dom+s442*X*dg1dom M4 = s441(om)*Y*dg1dom-s442*X*dg2dom K2DK1 = besselk(2,Y)/besselk(1,Y) I2DI1 = besseli(2,X)/besseli(1,X) I2DI1RK2DK1 = besseli(2,X)/besseli(1,X)*besselk(2,Y)/besselk(1,Y) *// L = NUMERATOR IN EQ.15* *// M = DENOMINATOR IN EQ.15* L = L1*K2DK1+L2*I2DI1+L3*1+L4*I2DI1RK2DK1 M = M1*K2DK1+M2*I2DI1+M3*1+M4*I2DI1RK2DK1 **G** = -L/M endfunction *//RESULTS* aa = 0 x0 = 2*%pi*0.7*10^5/v2*1.05 /*/STARTING POINT FOR WAVENUMBER k* *//SET UP A LOOP FOR EVALUATION THE PHASE AND GROUP* *//VELOCITIES AS A FUNCTION OF THE WAVE FREQUENCY f* for i = 1:1:77 f = 0.7*10^5+(i-1)*1*10^3 /*/WAVE FREQUENCY IN KHz* om = f*2*%pi /*/ANGULAR FREQUENCY* [xs,fxs,m] = fsolve(x0,T_Dysp_2,10^-12) k = xs /*/WAVENUMBER* vp = om/k /*/**PHASE VELOCITY* x = om y = k vgr = Group_Vel_2(x,y) /*/**GROUP VELOCITY* x0 = k*1.05 *//STORE THE RESULTS OF CALCULATIONS IN THE MATRIX aa* aa(i,1) = f/1000 aa(i,2) = vp aa(i,3) = vgr aa(i,4) = k end *//WRITE THE MATRIX aa INTO AN OUTPUT TXT FILE* write(’Torsional_2024_4.txt’,aa,’(e15.6,2x,e16.7,2x,e16.7,2x,e16.7)’)
